# Application of Statistical Methods in Predicting the Properties of Glass-Ceramic Materials Obtained from Inorganic Solid Waste

**DOI:** 10.3390/ma14102651

**Published:** 2021-05-18

**Authors:** Anna Zawada, Iwona Przerada, Małgorzata Lubas, Maciej Sitarz, Magdalena Leśniak

**Affiliations:** 1 Department of Materials Engineering, Faculty of Production Engineering and Materials Technology, Czestochowa University of Technology, 42-201 Czestochowa, Poland; iwona.przerada@pcz.pl (I.P.); malgorzata.lubas@pcz.pl (M.L.); 2 Department of Silicate Chemistry and Macromolecular Compounds, Faculty of Materials Science and Ceramics, AGH University of Science and Technology, 30 Mickiewicza Av., 30-059 Krakow, Poland; msitarz@agh.edu.pl (M.S.); mlesniak@agh.edu.pl (M.L.)

**Keywords:** experiment planning, statistical modeling, waste management, glass-ceramic materials

## Abstract

This paper uses mathematical methods as the basic tool at the stage of experiment planning. The importance of research programming applications was shown using the theory of experiments and the STATISTICA software. The method of experiment planning used in the case of studying the properties of a mixture, depending on its composition, features considerable complexity. The aim of the statistical analysis was to determine the influence of variable chemical composition of waste materials on selected properties of glass-ceramic materials. A statistical approach to multicomponent systems, such as ceramic sets, enables the selection of appropriate amounts of raw materials through the application of ‘a plan for mixtures’. To utilize the raw waste materials, e.g., slags from a solid waste incinerator, fly or bottom ashes, in the modeling of new materials, a mathematical relationship was developed, which enables estimating, based on the waste chemical composition, selected technological and practical properties of the glass so as to obtain a material featuring the required technological–practical parameters. For the obtained glasses, a comparative analysis of the experimentally and computationally determined properties was carried out: transformation temperature, liquidus temperature, density, and thermal expansion coefficient. The obtained high theoretical approximation (at the level of determination correlation coefficient R^2^ > 0.8) confirms the suitability of the polynomial model for mixtures for applications in the design of new glass-ceramic products.

## 1. Introduction

The accumulation of mineral residues in industrial production has reached a volume of several hundred-million tons/a year. Their recovery and/or disposal result in growing cost-intensive problems, such as a growth barrier or location problem [1]. Future legislative activities can be expected to further aggravate this situation [2]. Various attempts were made in the past to dispose of ashes and slags, e.g., from waste incineration [3,4,5,6].

The investigations carried out so far have shown that the production of higher-quality molded glass products from residues is most difficult because the composition of these substances, consisting of ashes, slags, and dusts, varies greatly [7,8,9,10,11]. In addition, melts of these materials usually have difficult processing properties, such as low viscosity, a strong tendency to crystallize, and a relatively high processing temperature. In preliminary experiments, it was shown that the material and processing properties could be adapted to the requirements by adding further residues, such as waste glass shards and old sands. The processing of residual waste in the melting phase into glass-ceramic (e.g., roofing material) and other shaped products requires the solution of material–technical and procedural issues [12,13,14]. Fluctuations in the composition of the residues to be used have an effect on both the product properties and the processing properties. So far, in glass technology, the composition has always been kept constant in order to ensure desired properties. In the recycling of residues, due to the strong fluctuations in their compositions in unfavorable cases, a high proportion of additional raw materials would be required, and thus, the amount of residual material decreases or the amount of product to be processed increases. In order to be able to use a maximum of residues, an attempt is made to produce products with the same properties but different compositions [15,16,17].

In each industrial sector, the use of solid waste is related to a permanent control of its chemical composition. In many cases, the variability of chemical composition of processed waste makes ensuring the stability of product properties obtained on its basis impossible.

Experimental methods are widely used in both research and industrial applications, although sometimes to achieve different goals. The primary goal of scientific research is to show that the influence of the chosen factor on the researcher of interest is statistically significant [18,19].

The task of modeling is different. In the given context, only the following subarea is interesting. There is a functional relationship between the influencing variables and the response quantity, which can be expressed quantitatively using, among other things, mathematical–statistical methods. A statistical model is then obtained which, in a limited area, reflects the relationships between influencing factors and target variables in more or less good approximation, depending on the quality of the adaptation. As a statistical model for influence and effect relationships, the regression model is often used [20,21].

The applicability of the model can be checked using various methods. There is a possibility of internal or external validation. In internal validation, the data are used both to build the model and to verify it. External validation is performed using other data which, however, must also be representative. To check the model, tests can also be carried out in which the target variables were predicted with the aid of the model. The measured values are compared with the predicted values. In the event of deviations, the reason must be found, and the model may have to be recreated. As a result, a model becomes better and better and more stable against unwanted influences [22,23].

The experimental methods are widely used both in science and in industrial applications. The basic objective of scientific research consists in showing that the influence of the selected factor on the magnitude, in which the researcher is interested, is statistically significant. The basic objective in the industry frequently consists in obtaining the maximum number of unbiased results, describing the factors affecting the production process, in addition to an as small as possible number of measurements.

Statistical test planning can be used for laboratory tests as well as for large-scale production tests. The aim of statistical experiment planning is to obtain a maximum of information with as few experiments as possible. However, there are restrictions. Extrapolations beyond the examined area are not permitted. The random scatter must also be taken into account. In systems with periodic or discontinuous behavior, statistical test planning can only be used to a limited extent.

Taking into consideration the basic chemical composition of the waste, as well as the role fulfilled by its individual components during the processing, it is possible to develop a computational model, based on which the main technological–practical parameters will be determined. The aluminosilicate glasses were the research area analyzed in this paper. In a multicomponent area, such as the chemical composition of glass, the matching of a computational model was based on a statistical plan for mixtures, starting from linear through to square and reaching a full third-order model. The choice of an appropriate model allowed to determine basic technological parameters is necessary to manufacture glass-ceramic materials. A controlled transformation of slags and fly ashes from solid waste incinerators into functional glass-ceramic materials allows limiting the storage of hazardous materials, protecting the environment from high-risk substances, and reducing the costs of their storage. Recycling such waste in combination with other waste materials, e.g., glass cullet, makes it possible to completely dispose of the end poisonous products of combustion [24,25].

Mathematical methods are most often used in the analysis of research results. This is the most appropriate and purposeful approach, but it should be stated that their application at this stage of research is too late. It is forgotten that mathematical methods should emerge as a basic tool in the planning phase of an experiment. The aim of this study is to pay special attention to the research phase, called planning the experiment.

## 2. Experimental Procedures

The purpose of each type of experimental research is to obtain information on the relationship between the values taken as input (independent variables) and the output values (dependent variables). This type of relationship is most often presented in the form of an approximating function of the research object; sometimes, the obtained relationship becomes a mathematical model of the research object. This happens when the obtained model is a cause-and-effect relationship based on the substantive analysis of the research object. Thus, not every approximating function of the research object obtained will be a mathematical model, but it can be used, for example, for optimization or simulation purposes. Another effect of the research may be the determination of the significance of the influence of the input quantities on the output quantities, which enables the elimination of irrelevant factors.

### 2.1. The Plan for Mixtures

The experiment planning allows answering two basic questions: how to plan the optimum experiment and how to analyze the obtained results of studies [18]. The method of experiment planning used in the case of studying the properties of a mixture, depending on its composition, features considerable complexity.

In the case of ceramic materials obtained and based on raw waste material, it is difficult to unequivocally characterize the area of practical applicability of such waste (ranges of chemical compositions and components’ quantitative ratios). The introduced amount of raw waste material is usually selected based mainly on its chemical composition, which sometimes varies in wide ranges.

A statistical approach to multicomponent systems, such as ceramic sets, enables—through the application of the ‘plan for mixtures’—selecting an appropriate amount of raw materials, so as to obtain a material featuring the required technological–practical parameters. Such plan can be used only if specific properties depend clearly on the amount of the introduced component of the mixture, i.e., of the set. Sometimes the components introduced to the set, even at small amounts, substantially change the properties, e.g., the addition causing the nucleation of the phase crystallizing in the glass [26].

**Figure 1:**
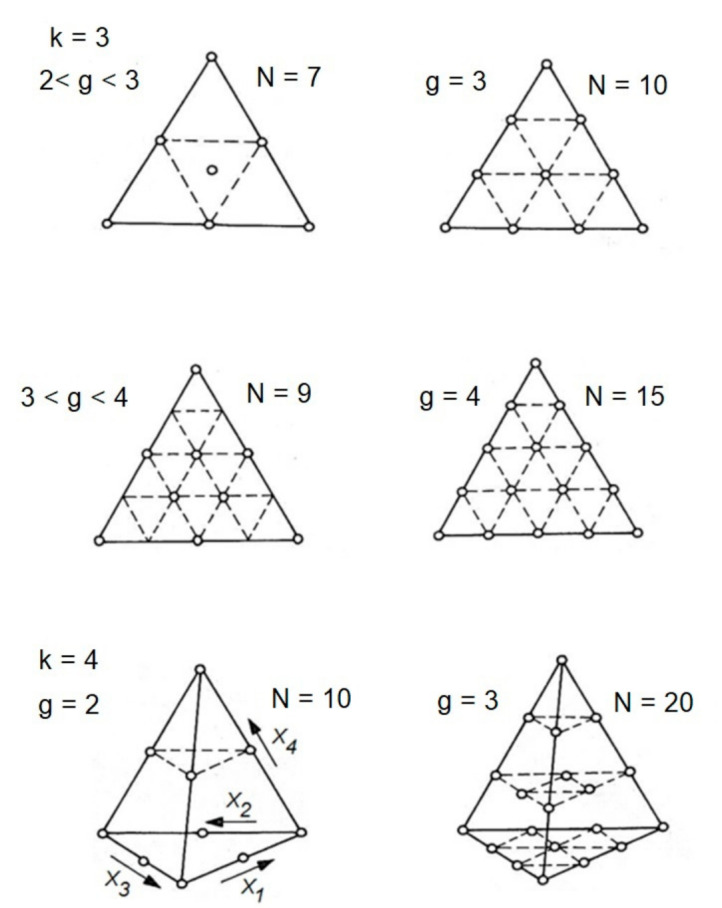
Number of experimental points for 3 and 4 components (k). N—number of experimental points; g—the degree of the regression polynomial [18].

Graphs in triangular coordinates are a widely used method to present shares in the mixture consisting most often of three components. A mixture of three components can be unequivocally determined by a point in a triangular coordinates system defined by these three variables. For each mixture, the total of individual component share values is 1. This means that in a mixture system, in which three components exist (k = 3), the experiment planning is limited to the area of a triangle. Then, it is necessary to carry out computations for mixtures of composition described by the coordinates of vertices (i.e., triangle corners) and of centers of gravity (triangle sides). Sometimes, such plans are supplemented by computations for a larger number of mixtures, in which compositions are described by coordinates of internal points within the triangle. At the increasing number of mixture components, the number of experimental points, situated in the spatial geometrical system in the plan of experiments, increases (Figure 1). From a mathematical point of view, the space of allowed values of x components of the mixture is a simple x with x vertices on an (x−1) dimensional hyperplane [4,18].

The number of measurement points N is determined by Equation (1):

(1) $$N=\left(\frac{g+k-1}{g}\right)=\frac{k\left(k+1\right)\left(k+2\right)\ldots \left(k+g-1\right)}{1\cdot 2\cdot \ldots \cdot g}$$

where k—the number of components, g—the degree of regression polynomial.

The properties described by experimental points in a triangle, or possibly in a polyhedron, can be presented by means of degree 2 ÷ 4 polynomial. Using the computations according to Equation (1), the minimum necessary number of experimental points was determined, which is also equal to the number of coefficients b of the regression polynomial (Table 1).

### 2.2. The Canonical Form of Polynomials for Mixtures

The matching of the polynomial model for mixtures, describing the matching of the response surface, starts from a linear model through square, special third degree and ending at a full third-degree model. Table 1 presents the number of coefficients for each model, depending on the number of mixture components.

The following models are usually used for mixtures: square and a simplified third degree. Equations (2)–(4) present the form of polynomials for these models in the case of three variables:

The linear model:

y = b_1_ × x_1_ + b_2_ × x_2_ + b_3_ × x_3_ (2)

The square model:

y = b_1_ × x_1_ + b_2_ × x_2_ + b_3_ × x_3_ + b_12_ × x_1_ × x_2_ + b_13_ × x_1_ × x_3_ + b_23_ × x_2_ × x_3_ (3)

The special cubic (third-degree) model:

y = b_1_ × x_1_ + b_2_ × x_2_ + b_3_ × x_3_ + b_12_ × x_1_ × x_2_ + b_13_ × x_1_ × x_3_ + b_23_ × x_2_ × x_3_ + b_123_ × x_1_ × x_2_ × x_3_ (4)

The response surface matching to the experimental results for mixtures is subject to limitations; this means that the sum of all components must be constant.

The analysis of experiments with mixtures in practice is based on a multiple (multidimensional) regression with a free term (b_0_) reduced to zero. The integrity condition for the mixture (the sum of all components is constant) can be satisfied by the use of multiple regression models, which do not contain a free term [3,18,27,28,29,30,31,32,33,34].

**Figure 2:**
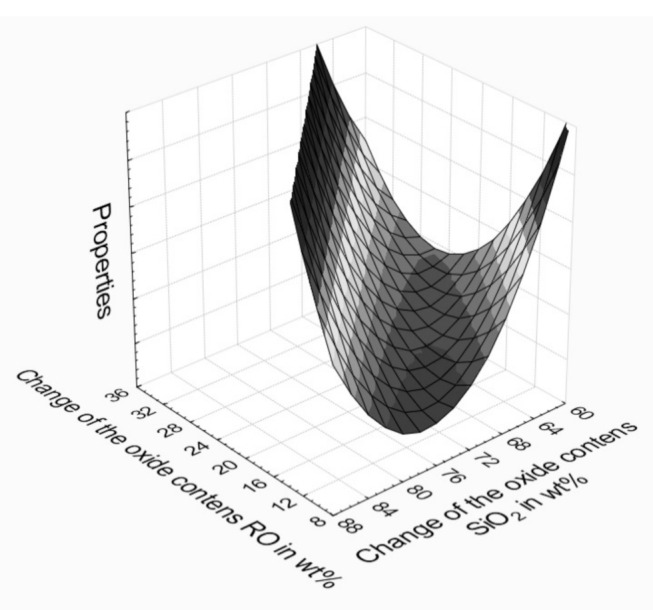
Changes in properties of aluminosilicate glasses related to their chemical composition (RO: CaO + MgO).

Analyzing the nature of glass properties changes, presented in model Figure 2 and obtained based on slags from the solid waste incineration, depending on the basic chemical composition, a second-order computational forecasting method in the form of Equation (3) is assumed [35,36]. All tested properties of glasses showed compliance with the adopted square model.

The adopted square model perfectly describes the area of studied glasses, classified as aluminosilicate glasses, featuring changes to properties depending on the chemical composition, which are not linear, such as those observed in the case of alkaline-silicate glasses.

## 3. Results and Discussions

To create a full picture of the development of secondary materials’ properties (the remainder originating at the solid waste incineration) depending on their chemical composition, percentage ranges of oxide contents, being the basis for further studies, have been estimated based on the literature data (Table 2).

Using a statistical research plan, compositions of 71 representative model glasses were selected from the SiO_2_-Al_2_O_3_-CaO-MgO-Fe_2_O_3_-R_2_O system, being the basis for further studies. For the determined oxide compositions of the glasses, sets were prepared from pure raw materials (CERTECH, Niedomice, Poland) and were then melted in corundum crucibles, in a laboratory furnace (CZYLOK, Jastrzębie-Zdrój, Poland), at a temperature of 1500 °C for 120 min, and then poured into a steel mold. Table 3 presents examples of nominal chemical compositions of melted glasses.

The following properties were determined experimentally for the obtained glasses: the transformation temperature—Tg, the liquidus temperature T_L_, density *ρ*, as well as the coefficient of thermal expansion *α*.

**Figure 3:**
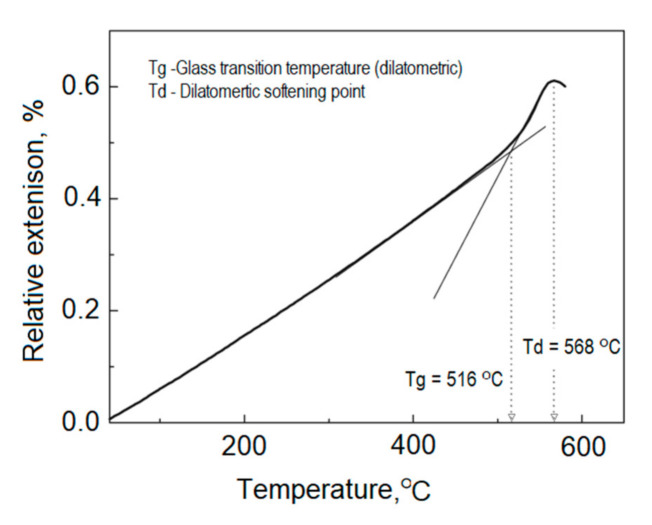
Measurement of Tg using dilatometry.

To determine the transformation temperature Tg (softening point), thermal expansion can be used, which is characterized by the average coefficient of linear expansion *α*. The change in the length of the glass sample during heating is recorded with a dilatometer. Below the Tg temperature, the glass expands linearly. Above Tg, the glass also expands linearly, but with a different slope of the straight line. The graphic designation of the Tg temperature is the place of the intersection of the tangents to the straight lines above and below the Tg (Figure 3).

The determination of thermal expansion coefficient *α* and transition temperature Tg is closely related to the cooling process [37,38]. Samples must be well cooled before measurement. For this, they are heated to a temperature of 30 K above Tg and then cooled to 150 K below Tg at a rate of 2 K/min. During the measurement, the heating rate should be 5 K/min. Figure 3 shows the measurement curve and its evaluation.

The determination of the thermal expansion coefficient and the transformation temperature Tg was carried out using the linear dilatometer DIL 402 C (Netzsch, Selb, Germany), under air atmosphere conditions, with a heating rate of 10 K/min.

The liquidus-TL temperature was determined in a gradient furnace with an accuracy of ±5 °C (gradient tube furnace in which you can create a temperature gradient across the furnace chamber (HTM Reetz GmbH, Berlin, Germany)). The samples are placed on platinum plates. The glass samples were annealed in the temperature range of 900–1250 °C. After the annealing process, the surface of the tested glasses was assessed using an optical microscope (Opta-Tech LAB–40M, Warsaw, Poland), and the liquidus temperature was determined on its basis.

In practice, the homogeneity and stability of the glass composition can be described by measuring the density, which is also an important factor for the calculation of other properties and design parameters. This parameter was determined for all samples by the hydrostatic weighing method (set for determining the density of solids and liquids, RADWAG, Radom, Poland). The density of the tested glasses ranged from 2.56 to 3.01 g/cm^3^.

**Figure 4:**
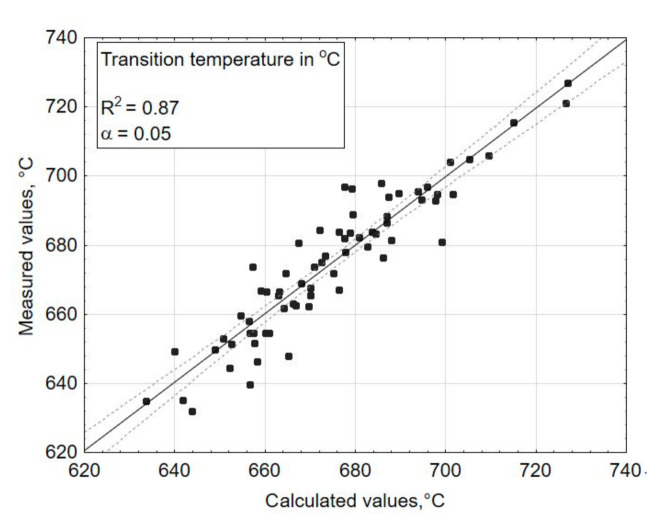
Comparison of the measured values (experimentally tested) with the estimated values (calculated using the chosen mathematical model)—for a dependent variable: transformation temperature (Tg) in °C.

**Figure 5:**
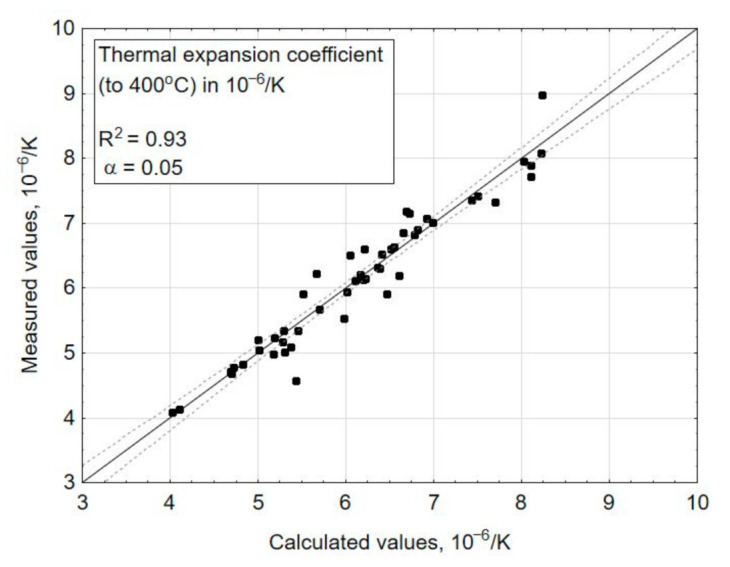
Comparison of the measured values (experimentally tested) and the estimated values (calculated using the chosen mathematical model)—for a dependent variable: thermal expansion coefficient *α*, in 10^−6^/K.

**Figure 6:**
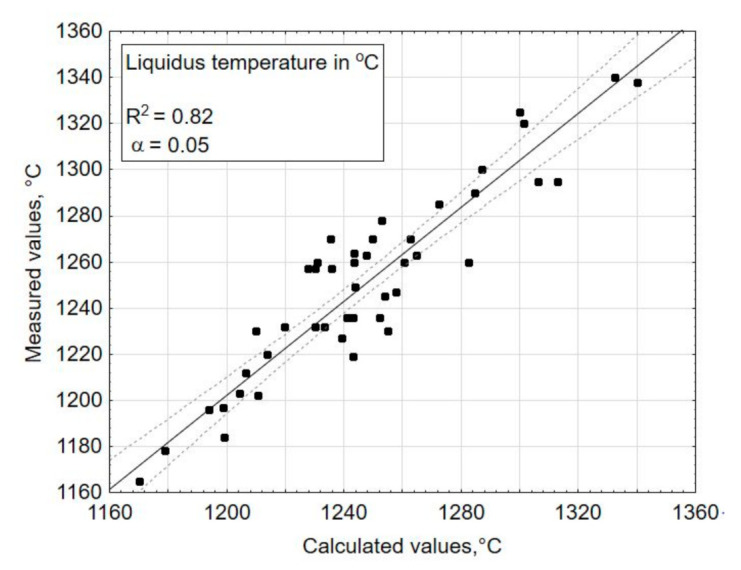
Comparison of the measured values (experimentally tested) and the estimated values (calculated using the chosen mathematical model)—for a dependent variable: liquidus temperature in °C.

**Figure 7:**
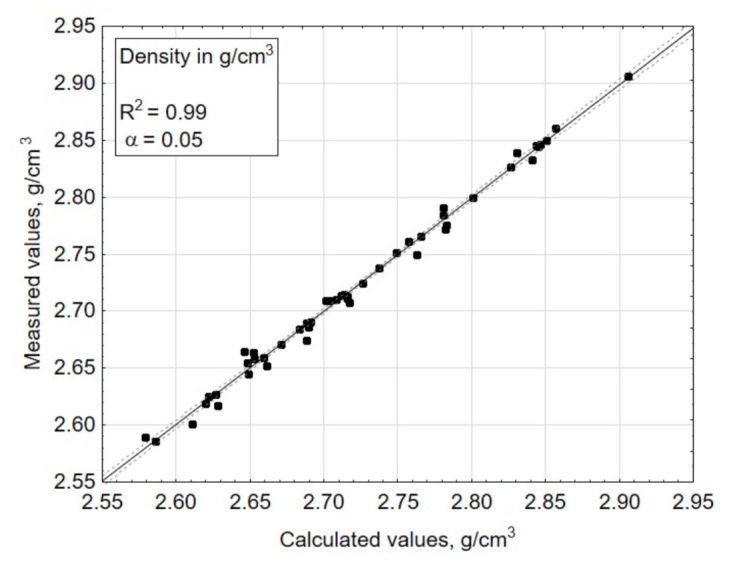
Comparison of the measured values (experimentally tested) and the estimated values (calculated using the chosen mathematical model)—for a dependent variable: density in g/cm^3^.

The multiple regression function (3), which fit the experimental data expressed by a determination correlation coefficient (R^2^), amounted to 0.87 for the transformation temperature—Tg (Figure 4), 0.93 for the coefficient of thermal expansion—*α* (Figure 5), 0.82 for the liquidus temperature—T_L_ (Figure 6), and 0.99 for the density (Figure 7). A poor fit of the experimentally measured and forecast values, in the case of liquidus temperature (the upper crystallization temperature), most likely resulted from the formation of various crystalline phases, for which additional parameters should be taken into account at the selection of coefficients b of Equation (3).

If the values result in a straight line after being entered in a probability network, they are normally distributed (Figure 5 and Figure 7). Individual values that are far from the straight line are likely to be outliers (Figure 6). There is no normal distribution if the values form a clearly curved line. In this case, the values should be transformed, e.g., calculated with the logarithmized or squared values. With the software (Statistica) used here for statistical test planning, the test of the normal distribution is carried out on the basis of the residuals, because the above procedure only applies to measured values determined under the same conditions, but here, each test point was only implemented once. The residuals result from the deviation between the estimated x value (calculated) and the measured value y.

R^2^ indicates how well the model fits the data and should be >0.8. For the selected parameters, the model fits the data well in all of the properties tested. R^2^ values range from 0.8 to 1.0.

## 4. Summary

A controlled processing of slags and fly ashes from a solid waste incineration plant into glass-crystalline materials allows reducing the dumping of hazardous substances, creating a major threat to environmental protection and increasing the related costs [39]. The recycling of such waste in combination with other waste materials, e.g., a cullet, provides the possibility of total disposal of the final products of the incineration.

Thanks to the results obtained in this work, it is possible to select an appropriate set of raw materials for a given chemical composition of glass. However, to predict individual property values from a given composition, or to calculate the composition for desired properties, it is necessary to know and consistently apply the underlying test plan and associated model.

To use waste in the manufacture of new materials, a mathematical relationship was determined. This allows estimating, based on the waste chemical composition, selected technological and practical properties of glass. The quality check of the created model confirmed that the model was well adapted to the data for almost all properties, and the predictability of the property values was either good or very good.

The conformity of computed and experimentally determined values indicates a possibility to apply such a model in practice, which enables theoretically characterizing the properties of glasses obtained on the basis of slags, ashes or dusts from a waste incinerator within a broad range. The developed model also enables forecasting the course of certain technological processes, e.g., crystallization. Such theoretical approximation is very useful, in particular when designing new glass-ceramic products in the ceramics industry.

## Figures and Tables

**Table 1:** Number of coefficients b of a regression polynomial in relation to the degree of the polynomial and the number of components [18].

Number of Components x	Regression Polynomial		
	2nd Degree	3rd Degree	4th Degree
	Number of Coefficients b		
3	6	10	15
4	10	20	35
5	15	35	70
6	21	56	126
7	28	84	210

**Table 2:** Oxides content ranges (in wt%).

**Oxide**	SiO_2_	Al_2_O_3_	CaO	MgO	Fe_2_O_3_	R_2_O *
**wt%**	45–60	8–20	10–25	3–15	2–10	4–6

* R—alkalimetals (Na, K).

**Table 3:** Examples of oxide compositions of glasses melted according to the research plan (wt%).

Nr	SiO_2_	Al_2_O_3_	CaO	MgO	Fe_2_O_3_	Alkali
1	46	21	23	2	2	5
2	44	13	21	8	9	4
3	59	9	10	13	2	5
4	46	10	22	14	2	5
5	60	19	11	3	4	4
6	45	21	23	2	3	5
7	58	14	19	2	3	4
8	45	23	18	3	6	4
9	46	19	10	10	7	5
10	43	21	18	3	10	4
11	44	8	21	4	4	6
12	46	9	19	3	4	7
13	45	9	19	4	4	6
11	43	8	21	4	3	6
12	44	9	19	4	4	6
13	45	8	19	4	4	7
14	54	8	16	3	3	6
15	46	9	17	3	4	7
16	49	8	18	3	3	7
17	48	8	18	3	4	7
18	48	8	18	3	4	7
19	48	8	19	3	4	7
20	47	8	18	3	4	7
14	50	8	18	3	4	7
15	46	9	20	4	4	7
16	47	9	20	4	4	7
21	47	9	20	4	4	7
22	47	8	19	4	4	7
23	48	8	19	3	4	7
24	46	8	19	3	4	7
25	51	8	18	3	4	7
26	51	8	18	3	4	6
27	48	8	20	3	4	6
28	45	8	19	4	4	6

## Data Availability

The data presented in this study are available on request from the corresponding author.
